# Association of early dietary fiber intake and mortality in septic patients with mechanical ventilation based on MIMIC IV 2.1 database: a cohort study

**DOI:** 10.1186/s12937-023-00894-1

**Published:** 2024-01-03

**Authors:** Xiaoyan Wang, Shuchuan Miao, Yuanwei Yang, Qilin Yang, Dejiao Meng, Hong Liang

**Affiliations:** 1 Department of Clinical Nutrition, The First Affiliated Hospital of Chengdu Medical College, Chengdu, Sichuan Province China; 2 Department of Neurosurgery, Chengdu Seventh People’s Hospital, Chengdu, Sichuan Province China; 3 Department of Intensive Care Unit, Affiliated Minshan Hospital of Chengdu Medical College, Ya’an, Sichuan Province China; 4 The Second Affiliated Hospital of Guangzhou Medical University, Guangzhou, Guangdong China; 5 Department of Intensive Care Unit, Nanjing First Hospital, Nanjing Medical University, Nanjing, Jiangsu Province China

**Keywords:** Dietary fiber, Fiber index, Sepsis, Critical care, Critical illness, Mortality

## Abstract

**Background:**

Whether early dietary fiber intake in septic patients is associated with a better clinical prognosis remains unclear, especially the time and the amount. Therefore, we assessed the association between early dietary fiber intake and clinical outcomes in septic patients by examining an extensive database.

**Methods:**

We conducted a retrospective cohort study using data from the MIMIC IV 2.1 database, focusing on consecutive septic patients requiring mechanical ventilation in medical or mixed medical-surgical ICUs. We collected patient demographics and nutritional data. Dietary fiber amounts were calculated according to enteral nutrition instructions from manufacturers within the first 72 h after admission. After adjusting for covariates, we employed restricted cubic spline (RCS) regression to investigate the relationship between fiber intake (FI) and 28-day mortality. Patients were categorized into three groups based on their fiber index (FI) within 72 h of admission: low fiber index (LFI) group when FI was < 3 g/(%), medium fiber index (MFI) group when FI ranged from 3 to 35 g(%), and high fiber index (HFI) group when FI ≥ 35 g(%). Univariate and multivariate Cox proportional hazards regression models were utilized to assess the association between early FI and 28-day mortality. We ultimately employed Kaplan–Meier (KM) curves and log-rank test visually represent the association between FI and 90-day mortality. The second outcomes include ICU-acquired infections and the hospital and ICU death, length of hospital and ICU stay, and length of mechanical ventilation.

**Results:**

Among 1057 subjects, 562 (53.2%) were male, with a median age of 64.8 years (IQR 53.4–75.2). We observed a J-shaped relationship between FI and 28-day mortality. The MFI group exhibited the lowest 28-day mortality [adjusted HR 0.64 (0.45–0.91), *p* = 0.013] and the lowest rate of hospital mortality [adjusted OR 0.60 (0.39–0.93), *p* = 0.022], with no statistically significant differences noted in the HFI group when compared to the LFI group. Similar patterns were observed for 60-day and 90-day mortality. However, no statistically significant differences were observed in other secondary outcomes after adjusting for covariates.

**Conclusion:**

Early medium fiber index intake improved 28-day mortality and lower hospital mortality in septic M/SICU patients on mechanical ventilation.

**Supplementary Information:**

The online version contains supplementary material available at 10.1186/s12937-023-00894-1.

## Introduction

The dietary fiber (DF) required by critically ill patients remains unknown, including when and the amount. Studies have shown DF can reduce diarrhea [1–5], improve intestinal motility [6], and also with safety and tolerability profile in hemodynamically stable critically ill patients, though rare studies reported [7] the fatal complications. The current Clinical Nutrition guidelines [8, 9] for critical illness have no explicit recommendation about DF use, and there are no recommendations about using nutritional formulas containing DF or supplements for DF at the early stage of sepsis.

Sepsis is one of the most common illnesses in the ICU, with its leading cause of morbidity and mortality worldwide [10] and one of the most costly diseases [11]. Early enteral nutrition for critical patients is vital in maintaining gut function. Widely published literature has presented DFs' indirect anti-inflammatory effects in healthy and hospitalized patients [2, 12], and a few reports have reported the effects of probiotics or synbiotics in critical patients [13, 14]. However, little was known about DF in septic patients, especially the association between the amount of early DF intake and the clinical outcomes.

Differing opinions on DF and its impact on mortality and other clinical outcomes can be attributed to variations in DF type, quantity, and duration of DF used [14–16]. Studies in critical care patients have examined DF from various sources, including symbiotic, enteral nutritional formula, and DF supplements. Our study specifically focused on soluble DF from enteral nutritional formulas, as it provided a consistent amount. Notably, Fu et al. [17]. Found that higher fiber intake, as measured by the Fiber Index (FI), was associated with increased production of short-chain fatty acids (SCAFs) and was well-tolerated by critical patients within 72 h of ICU admission.

Therefore, the objective of this study was to evaluate whether DF intake 72 h after admission had a relationship between the amount (measure by FI) and clinical outcomes.

## Methods

### Patients

**Fig. 1:**
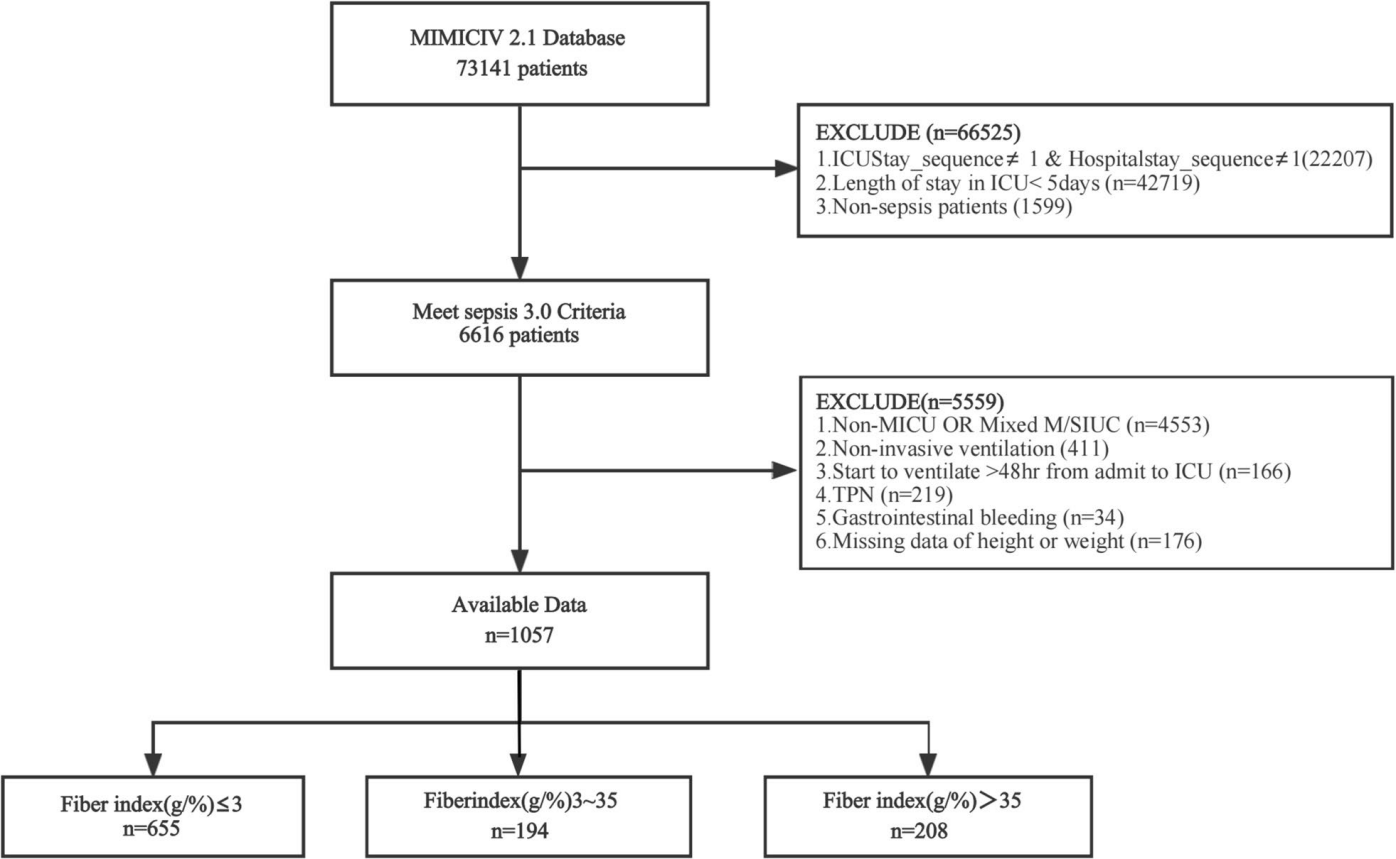
Flowchart of the study

Patients were eligible for analysis as below: 1. Critically ill adults meet the criteria of sepsis 3.0 definition [18], the clinical criteria is as below: suspected or documented infection and an acute increase of ≥ 2 SOFA points (a proxy for organ dysfunction) [18]; 2. Be in hospital and be in ICU for the first time; 3. ICU stay lasting ≥ 5 days; 4. Receipt of invasive mechanical ventilation initiated within 48 h of ICU admission; 5. Absence of gastrointestinal bleeding; 6. Receiving nutrition support, either enteral nutrition [EN] OR EN plus parenteral nutrition [PN]); 7. Nutritional variables are consecutive. Patients with missing weight, height, or caloric intake data were excluded, as shown in Fig. 1 in the flowchart (Fig. 1).

Baseline characteristics include age, gender, Body Mass Index (BMI), Sequential Organ Failure Assessment (SOFA), Simplified Acute Physiology Score II(SASPII), Charlson Comorbidity Index (CCI), number of antibiotics within 72 h, norepinephrine equivalents within 72 h, proton pump inhibitors or H_2_ receptor antagonists within 72 h.

### Nutrition data

The calculation of energy intake encompasses both non-nutritional and nutritional sources, including substances like propofol, glucose infusions, enteral nutrition formulas, and parenteral nutrition. The target energy was determined by considering corrected ideal body weight, age, and gender (the target energy is based on the calculation of the fiber index) [19, 20]. Feeding route, whether early enteral nutrition (feeding within 48 h of admit to ICU), energy achievement (the ratio of actual energy to target energy), actual non-nutrition energy, enteral nutrition energy, protein, fiber intake and FI were collected.

### Fiber index and formula

We checked the DF amount from the database of enteral nutrition instructions from different manufacturers (as shown in sTable1.xlsx). The amount of fiber consumed depends on total caloric consumption, so the relative consumption of fiber was a calorie-corrected “fiber index” [17]. We use FI [17] as fiber intake over the 72 h divided by the percentage of target energy received, the calculation formula is as follows:

$$Fiber\;index\left(FI\left(g/\left(\%\right)\right)\right)=\frac{Total\;Fiber\;intake(g)(within\;72hr)}{\frac{Actual\;energy\;intake(kcal)}{Target\;energy\;intake(kcal)}\;(\%)(within\;72hr)}$$

**Fig. 2:**
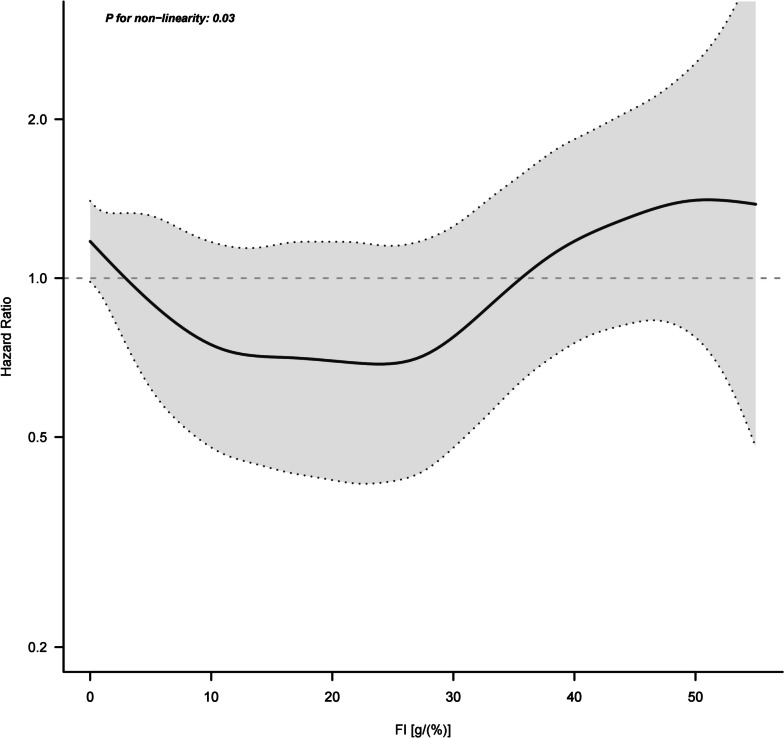
Relationship between FI and 28-day mortality. Model adjusted for age, BMI, SOFA, SAPS II, CCI, vasoactive agents, norepinephrine equivalents, feeding route and early enteral nutrition

Patients were stratified into three groups based on their 72-h Fiber Index (FI). Using Restricted Cubic Spline (RCS) regression analysis, we identified the lowest hazard ratio (HR) when the FI ranged from 3 to 35 g/(%). Accordingly, we established cutoff values of 3 and 35 (see Fig. 2). These categories were defined as follows: the Low Fiber Index (LFI) group for FI < 3 g/(%) (including FI = 0), the Medium Fiber Index (MFI) group for FI ranging between 3 and 35 g/(%), and the High Fiber Index (HFI) group for FI ≥ 35 g/(%).

### Outcome data

We designated the first 72 h following admission to the ICU as the ‘early’ phase. We assessed patient mortality at four time points: 28 days, 60 days, 90 days, and one year after admission. Hospital/ICU mortality was defined as the occurrence of death within the hospital or ICU setting. Length of ICU/hospital defined as the duration of the patient’s stay in either the ICU or the hospital. The following formula of Norepinephrine equivalents in ICU settings (all in mcg/kg/min, except vasopressin in units/min): Norepinephrine equivalents = norepinephrine + epinephrine + phenylephrine/10 + dopamine/100 + metaraminol/8 + vasopressin*2.5 + angiotensin II*10 [21]. Lastly, the length of mechanical ventilation represented the duration of invasive mechanical ventilation. Infection complications were defined as follows: (1) Ventilator-associated pneumonia was defined as a new pneumonia that develops after 48 h of endotracheal intubation [22, 23]. The data was extracted from the patient’s diagnosis according to the ICD-9 code (ICD-9 99731 Ventilator associated pneumonia). (2) Clostridium difficile infection was defined as infection by Clostridium difficile. The data was extracted from the patient’s diagnosis according to the ICD-9 and ICD10 code (ICD-9 00845, ICD10 A047, A0471, A0472 Intestinal infection due to Clostridium difficile). (3) Early-onset nosocomial infection was defined as an infection occurring within 48 to 120 h after admission, involving different microorganisms than those present at the time of admission, we extracted from the microorganism information to identify the new different microorganism. The same way to identify late-onset nosocomial infection, which are characterized by the emergence of new microorganisms after 120 h of admission [24].

### Study description

A retrospective cohort study was conducted among all consecutive, septic, invasive mechanically ventilated patients in a mixed medical-surgical or medical ICU from MIMIC IV 2.1 database.

### Data collection

Data for this study were sourced from the Multiparameter Intelligent Monitoring in Intensive Care IV 2.1 database, with data extraction carried out using PostgreSQL v11.5.

### Statistical analysis

Demographic variables and nutritional data for the initial 72 h were compared among groups. Continuous variables are presented as either mean ± standard deviation (SD) or median (interquartile range, IQR) and were assessed for group differences using one-way ANOVA, with multiple comparisons conducted using the SNK method. Categorical variables are presented as counts or percentages and were compared among groups using the chi-squared test.

We conducted Restricted Cubic Spline (RCS) regression analysis with four knots placed at the 5th, 35th, 65th, and 95th percentiles of FI, adjusting for variables in model 1. This analysis aimed to evaluate nonlinearity and explore the dose–response relationship between FI and HR of 28-day mortality. Our findings revealed that the FI range of 3 ~ 35 g/(%) was associated with the lowest hazard ratio (HR), leading us to select 3 and 35 as the cutoff values, as depicted in Fig. 2.

Univariate and multivariate Cox proportional hazards regression models were use to evaluate the relationship between early FI and the primary outcomes. Model 1 underwent full adjustment, including confounders such as age, BMI, SOFA, SAPS II, CCI, vasoactive agents, norepinephrine equivalents, feeding route, and early enteral nutrition and actual energy intake. Survival analysis was performed using Kaplan–Meier (KM) curves and the log-rank test. Second outcomes were assessed by multivariate cox regression analysis, multivariate logistic regression analysis, and multivariate linear regression analysis.

We employed multivariate regression to elucidate the association between FI and 28-day mortality. The model included factors meeting two criteria: (1) statistical significance with a *p*-value < 0.05 in univariate regression analysis, and (2) clinical relevance to the outcome.

To robust of our findings, we performed subgroup analyses, potential modifications of the relationship between FI and 28-day mortality were assessed, including the following variables: age (< 65,65 ~ 80 and ≥ 80 years), BMI (< 18.5, 18.5 ~ 25, 25 ~ 30, and ≥ 30 kg/m^2^), SOFA score (< 6 vs. ≥ 6), feeding route(EN vs. EN + PN), vasoactive agents used(NO vs. Yes), number of antibiotics (< 3 vs. ≥ 3).

All analyses were conducted using the statistical software packages R (http://www.R-project.org, The R Foundation) and Free Statistics Software version 1.7, with significance defined as *p* < 0.05.

## Results

### Patients’ characteristics and 72 h nutrition data

As depicted in Fig. 1, a total of 1057 subjects were included in the final analysis. None of the subjects received fibrin supplements or probiotics while in the ICU. Among these 1057 subjects, 562 (53.169%) were male, with a median age of 64.8 (IQR 53.4, 75.2) years. There were no statistically significant differences among the groups with respect to age, BMI, SASPII, CCI, the number of antibiotics administered, or the use of PPI or H_2_RA medications. However, there was variation in sex distribution among the groups. The MFI group had a higher proportion of female subjects, while the HFI group had a higher proportion of male subjects. Notably, the SOFA score was higher in the LFI group compared to the other groups (*p* = 0.046). Additionally, the HFI group had the highest number of patients who did not require vasoactive agents [LFI vs. MFI vs. HFI: 182 (27.786%) vs. 67 (34.536%) vs. 80 (38.462%), *p* = 0.008], as well as the highest norepinephrine equivalents (see Table 1).

**Table 1:** Baseline demographic and clinical characteristics among groups

Variables	Total (*n* = 1057)	Group by Fiber index (g/(%))			*P* value
		**LFI group** **(*n* = 655)**	**MFI group** **(*n* = 194)**	**HFI group** **(*n* = 208)**	
**General Characteristics**					
**Sex (Male)**	562 (53.169)	349 (53.282)	88 (45.361)	125 (60.096)	0.013
**Age(year)**	64.8 (53.4, 75.2)	64.6 (52.9, 75.3)	64.5 (53.3, 74.4)	66.2 (55.4, 75.2)	0.52
**Age(year)**					0.702
< 65	535 (50.615)	337 (51.45)	99 (51.031)	99 (47.596)	
65 ~ 80	359 (33.964)	213 (32.519)	68 (35.052)	78 (37.5)	
≥ 80	163 (15.421)	105 (16.031)	27 (13.918)	31 (14.904)	
**BMI (kg/m**^**2**^**)**	28.1 (23.7, 34.2)	28.5 (24.0, 34.6)	27.4 (23.4, 33.3)	27.4 (23.4, 33.0)	0.196
**BMI (kg/m**^**2**^**)**					0.66
< 18.5	37 (3.500)	20 (3.053)	7 (3.608)	10 (4.808)	
18.5 ~ 25	298 (28.193)	181 (27.634)	58 (29.897)	59 (28.365)	
25 ~ 30	290 (27.436)	174 (26.565)	53 (27.32)	63 (30.288)	
≥ 30	432 (40.870)	280 (42.748)	76 (39.175)	76 (36.538)	
**Scores in ICU**					
**SOFA**	10.0 (7.0, 13.0)	10.0 (8.0, 13.0)	10.0 (7.0, 12.0)	10.0 (7.0, 12.0)	0.046
**SASPII**	45.0 (35.0, 56.0)	46.0 (36.0, 58.0)	43.0 (34.0, 53.0)	44.0 (35.0, 55.0)	0.051
**CCI**	6.0 (4.0, 8.0)	6.0 (4.0, 8.0)	5.0 (3.0, 8.0)	6.0 (4.0, 8.0)	0.581
**Medicine use within 72 h**					
**Number of antibiotics**	3.0 (2.0, 4.0)	3.0 (2.0, 4.0)	3.0 (2.0, 4.0)	3.0 (2.0, 4.0)	0.315
**Number of antibiotics**					0.819
< 3	335 (31.693)	209 (31.908)	58 (29.897)	68 (32.692)	
≥ 3	722 (68.307)	446 (68.092)	136 (70.103)	140 (67.308)	
**Norepinephrine equivalents**	0.1 (0.0, 1.0)	0.1 (0.0, 1.7)	0.1 (0.0, 0.3)	0.1 (0.0, 0.2)	< 0.001
**Vasoactive agents**					0.008
No	329 (31.126)	182 (27.786)	67 (34.536)	80 (38.462)	
Yes	728 (68.874)	473 (72.214)	127 (65.464)	128 (61.538)	
**PPI OR H**_**2**_**RA**					0.942
No	37 (3.500)	22 (3.359)	7 (3.608)	8 (3.846)	
Yes	1020 (96.500)	633 (96.641)	187 (96.392)	200 (96.154)	

Values are n (%) or median (25th–75th percentile)

*Abbreviations*: *BMI* Body Mass Index, *SOFA* Sequential Organ Failure Assessment, *SASPII* Simplified Acute Physiology Score II, *CCI* Charlson comorbidity index, *PPI* Proton pump inhibitors, *H*_*2*_*RA* H_2_ receptor antagonists

Regarding nutritional variables over the initial 72 h, several notable differences showed among the groups. The LFI group had the highest prevalence of patients receiving enteral nutrition plus parenteral nutrition (EN + PN) as their feeding route [LFI vs. MFI vs. HFI: 62 (9.466%) vs. 7 (3.608%) vs. 9 (4.327%), *p* = 0.004]. Conversely, the HFI group exhibited the highest proportion of patients receiving early enteral nutrition (EEN) [LFI vs. MFI vs. HFI: 211 (32.214%) vs. 102 (52.577%) vs. 167 (80.288%), *p* < 0.001]. Additionally, the HFI group demonstrated the highest energy intake within the initial 72 h [LFI vs. MFI vs. HFI: 0.2 (IQR: 0.1, 0.4) vs. 0.2 (IQR: 0.2, 0.4) vs. 0.4 (IQR: 0.2, 0.5), *p* < 0.001], as well as the highest actual enteral nutrition energy intake during the same period [LFI vs. MFI vs. HFI: 0.0 (IQR: 0.0, 776.4) vs. 363.9 (IQR: 197.7, 866.9) vs. 1578.0 (IQR: 841.8, 2394.0), *p* < 0.001]. Furthermore, the HFI group exhibited the highest actual protein intake [LFI vs. MFI vs. HFI: 0.0 (IQR: 0.0, 41.2) vs. 19.6 (IQR: 10.1, 42.4) vs. 79.9 (IQR: 41.7, 117.4), *p* < 0.001]. Moreover, the HFI group exhibited the highest actual fiber intake [LFI vs. MFI vs. HFI: 0.0 (IQR: 0.0, 0.0) vs. 3.9 (IQR: 2.4, 7.1) vs. 21.6 (IQR: 11.8, 31.8), *p* < 0.001], and the highest Fiber Index (FI) [LFI vs. MFI vs. HFI: 0.0 (IQR: 0.0, 0.0) vs. 17.4 (IQR: 10.2, 27.3) vs. 55.5 (IQR: 42.7, 68.5), *p* < 0.001] (see Table 2).

**Table 2:** Baseline nutritic characteristics within 72 h in ICU among groups

Variables	Total (*n* = 1057)	Group by Fiber index (g/(%))			*P* value
		**LFI group** **(*n* = 655)**	**MFI group** **(*n* = 194)**	**HFI group** **(*n* = 208)**	
**Feeding route: EN**	979 (92.621)	593 (90.534)	187 (96.392)	199 (95.673)	0.004
**Feeding route: EN + PN**	78 (7.379)	62 (9.466)	7 (3.608)	9 (4.327)	
**Early enteral nutrition: NO**	577 (54.588)	444 (67.786)	92 (47.423)	41 (19.712)	< 0.001
**Early enteral nutrition: Yes**	480 (45.412)	211 (32.214)	102 (52.577)	167 (80.288)	
Target Energy(kcal)^*^	6152.0 (4529.0, 7622.0)	6152.0 (4718.0, 7916.0)	5532.0 (4293.0, 7353.0)	6442.0 (4765.0, 7695.0)	0.008
**Actual energy intake(kcal)**	1507.0 (809.4, 2558.0)	1283.0 (696.5, 2322.0)	1427.0 (874.6, 2254.0)	2252.0 (1501.0, 3368.0)	< 0.001
**Energy achievement**	0.2 (0.1, 0.4)	0.2 (0.1, 0.4)	0.2 (0.2, 0.4)	0.4 (0.2, 0.5)	< 0.001
**Actual protein intake(g)**	15.6 (0.0, 72.4)	0.0 (0.0, 41.2)	19.6 (10.1, 42.4)	79.9 (41.7, 117.4)	< 0.001
**Actual non-nutrient energy intake(kcal)**	373.5 (0.0, 1437.0)	90.0 (0.0, 840.2)	400.7 (203.2, 990.6)	1578.0 (869.6, 2434.0)	< 0.001
**Actual enteral nutrition energy intake(kcal)**	324.0 (0.0, 1385.0)	0.0 (0.0, 776.4)	363.9 (197.7, 866.9)	1578.0 (841.8, 2394.0)	< 0.001
**Actual fiber intake(g)**	0.0 (0.0, 5.4)	0.0 (0.0, 0.0)	3.9 (2.4, 7.1)	21.6 (11.8, 31.8)	< 0.001
**Fiber index(g/(%))**	0.0 (0.0, 26.1)	0.0 (0.0, 0.0)	17.4 (10.2, 27.3)	55.5 (42.7, 68.5)	< 0.001

Values are n (%) or median (25th–75th percentile)

*Abbreviations*: *EN* Enteral nutrition, *PN* Parenteral Nutrition, *FI* Fiber Index, *LFI* Low fiber index(FI < 3 g/(%)), *MFI* Medium fiber index(3 ≤ FI <35 g/(%), *HFI* High fiber index(≥ 35 g/(%)), *IQR* Interquartile range, Actual non-nutrient energy intake: include energy of dextrose and propofol. ^*^The target energy is based on the calculation of the fiber index

### Primary outcome

Univariate Cox proportional hazards analysis identified several factors significantly affecting 28-day mortality, including age, BMI, SOFA, SAPS II, CCI, vasoactive agents administered, average norepinephrine equivalents, and actual energy intake within 72 h. Specifically, the MFI group exhibited a HR of 0.63 with a 95% CI of (0.45 ~ 0.89) compared to the LFI group (Table 3). After adjusting for covariates, the results for 28-day mortality consistently revealed the lowest HR of 0.64 with a 95% CI of (0.45 ~ 0.91) compared to the LFI group, but the HFI group showed no statists different compared to the LFI group. Similar outcomes remained in 60 and 90-day mortality in MFI group (See Table 4).

**Table 3:** Univariate Cox regression models for 28- day mortality

Predictors	HR (95%CI)	*P* (Wald’s test)	*P* (LR-test)
Fiber Index[g/(%)] < 3 ref			0.009
3 ~ 35	0.63 (0.45,0.89)	0.008	
≥ 35	1.1 (0.84,1.44)	0.502	
Sex: Female vs Male	1.01 (0.81,1.27)	0.921	0.921
Age (year)	1.03 (1.02,1.04)	< 0.001	< 0.001
BMI (kg/m^2^)	0.99 (0.97,1)	0.04	0.034
SOFA	1.07 (1.04,1.1)	< 0.001	< 0.001
SAPSII	1.02 (1.01,1.02)	< 0.001	< 0.001
CCI	1.16 (1.12,1.2)	< 0.001	< 0.001
Number of antibiotics within 72 h	1.0083 (0.9337,1.0889)	0.833	0.834
Vasoactive agents within 72 h: Yes vs. No	1.7 (1.3,2.23)	< 0.001	< 0.001
Average norepinephrine equivalents within 72 h	1.09 (1.04,1.13)	< 0.001	< 0.001
PPI OR H_2_RA used within 72 h: Yes vs. No	1.94 (0.86,4.35)	0.108	0.072
Feeding route: PN + EN vs. EN	0.8 (0.51,1.25)	0.324	0.307
Early enteral nutrition: Yes vs. No	0.87 (0.69,1.09)	0.223	0.222
Actual energy intake within 72 h (kcal)	0.9998 (0.9997,0.9999)	0.001	< 0.001

*Abbreviations*: *HR* Hazard Ratio, *CI* Confidence interval, *BMI* Body Mass Index, *SOFA* Sequential Organ Failure Assessment, *SASPII* Simplified Acute Physiology Score II, *CCI* Charlson comorbidity index, *PPI* Proton pump inhibitors, *H*_*2*_*RA* H_2_ receptor antagonists, *EN* Enteral nutrition, *PN* Parenteral Nutrition, Actual energy intake include PN, EN and non-nutrient energy intake (dextrose and propofol)

**Table 4:** Primary and second outcomes

		**Non-adjust Model**		**Model 1**	
	N (%)	HR/OR/β(95%CI)	*p* value	HR/OR/β(95%CI)	*P* value
**Primary outcomes**					
**28-day mortality, n (%)**					
LFI Group *n* = 655	199 (30.4)	1(Ref)		1(Ref)	
MFI Group *n* = 194	39(20.1)	0.63 (0.45 ~ 0.89)	0.008	0.64 (0.45 ~ 0.91)	0.011
HFI Group *n* = 208	69(33.2)	1.1 (0.84 ~ 1.44)	0.502	1.18 (0.87 ~ 1.62)	0.291
**60-day mortality, n (%)**					
LFI Group *n* = 655	231 (35.3)	1(Ref)		1(Ref)	
MFI Group *n* = 194	50 (25.8)	0.69 (0.51 ~ 0.93)	0.016	0.69(0.51 ~ 0.95)	0.022
HFI Group *n* = 208	83 (39.9)	1.15 (0.89 ~ 1.47)	0.29	1.24 (0.93 ~ 1.66)	0.137
**90-day mortality, n (%)**					
LFI Group *n* = 655	251 (38.3)	1(Ref)		1(Ref)	
MFI Group *n* = 194	56 (28.9)	0.7 (0.53 ~ 0.94)	0.017	0.72 (0.54 ~ 0.97)	0.031
HFI Group *n* = 208	86 (41.3)	1.1 (0.86 ~ 1.4)	0.462	1.18 (0.92 ~ 1.60)	0.18
**1-year mortality, n (%)**					
LFI Group *n* = 655	298 (45.5)	1(Ref)		1(Ref)	
MFI Group *n* = 194	75 (38.7)	0.79 (0.61 ~ 1.01)	0.065	0.79 (0.61 ~ 1.02)	0.074
HFI Group *n* = 208	108 (51.9)	1.18 (0.94 ~ 1.47)	0.147	1.26 (0.98 ~ 1.63)	0.066
**Second Outcomes**					
**Hospital death**					
LFI Group *n* = 655	184 (28.1)	1(Ref)		1(Ref)	
MFI Group *n* = 194	36 (18.6)	0.58 (0.39 ~ 0.87)	0.008	0.60 (0.39 ~ 0.93)	0.022
HFI Group *n* = 208	59 (28.4)	1.01 (0.72 ~ 1.43)	0.939	1.25 (0.83 ~ 1.87)	0.292
**ICU death**					
LFI Group *n* = 655	151 (23.1)	1(Ref)		1(Ref)	
MFI Group *n* = 194	31 (16)	0.63 (0.42 ~ 0.97)	0.036	0.67 (0.43 ~ 1.05)	0.080
HFI Group *n* = 208	47 (22.6)	0.97 (0.67 ~ 1.41)	0.891	1.12 (0.73 ~ 1.72)	0.592
**Length of hospital saty**					
LFI Group *n* = 655		0(Ref)		0(Ref)	
MFI Group *n* = 194		-2.18 (-4.59 ~ 0.22)	0.076	-0.96 (-3.33 ~ 1.40)	0.425
HFI Group *n* = 208		-3.09 (-5.43 ~ -0.75)	0.01	-1.36 (-3.82 ~ 1.10)	0.281
**Length of ICU saty**					
LFI Group *n* = 655		0(Ref)		0(Ref)	
MFI Group *n* = 194		-1.52 (-2.77 ~ -0.27)	0.017	-0.84 (-2.06 ~ 0.38)	0.176
HFI Group *n* = 208		-1.84 (-3.05 ~ -0.62)	0.003	-0.95 (-2.22 ~ 0.31)	0.14
**Length of Mechanical Ventilation**					
LFI Group *n* = 655		0(Ref)		0(Ref)	
MFI Group *n* = 194		-0.93 (-1.74 ~ -0.13)	0.023	-0.47 (-1.26 ~ 0.32)	0.247
HFI Group *n* = 208		-0.77 (-1.56 ~ 0.01)	0.053	-0.16 (-0.99 ~ 0.66)	0.695
**Ventilator-associated pneumonia**					
LFI Group *n* = 655	140 (21.4)	1(Ref)		1(Ref)	
MFI Group *n* = 194	38 (19.6)	0.9 (0.6 ~ 1.34)	0.591	0.98 (0.65 ~ 1.49)	0.939
HFI Group *n* = 208	43 (20.7)	0.96 (0.65 ~ 1.41)	0.829	1.04 (0.68 ~ 1.60)	0.841
**Early Nosocomial infections**					
LFI Group *n* = 655	246 (37.6)	1(Ref)		1(Ref)	
MFI Group *n* = 194	66 (34)	0.86 (0.61 ~ 1.2)	0.37	0.92 (0.65 ~ 1.31)	0.655
HFI Group *n* = 208	67 (32.2)	0.79 (0.57 ~ 1.1)	0.163	0.89 (0.62 ~ 1.27)	0.51
**Late Nosocomial infections**					
LFI Group *n* = 655	335 (51.1)	1(Ref)		1(Ref)	
MFI Group *n* = 194	80 (41.2)	0.67 (0.48 ~ 0.93)	0.016	0.75 (0.53 ~ 1.05)	0.090
HFI Group *n* = 208	90 (43.3)	0.73 (0.53 ~ 1)	0.048	0.87 (0.61 ~ 1.23)	0.421
**Clostridium difficile infection**					
LFI Group *n* = 655	25 (3.8)	1(Ref)		1(Ref)	
MFI Group *n* = 194	5 (2.6)	0.67 (0.25 ~ 1.77)	0.414	0.74 (0.27 ~ 2.00)	0.553
HFI Group *n* = 208	10 (4.8)	1.27 (0.6 ~ 2.7)	0.529	1.62 (0.70 ~ 3.78)	0.262

Model 1: Adjust for age, BMI, SOFA, SAPSII, CCI, Vasoactive agents, Norepinephrine equivalents, feeding route, Early enteral nutrition and actual energy intake. Actual energy intake includes PN, EN and non-nutrient energy intake (dextrose and propofol)

*Abbreviations*: *HR* Hazard ratio, *OR* Odds ratio, *CI* Confidence interval, *BMI* Body Mass Index, *SOFA* Sequential Organ Failure Assessment, *SASPII* Simplified Acute Physiology Score II, *CCI* Charlson comorbidity index

**Fig. 3:**
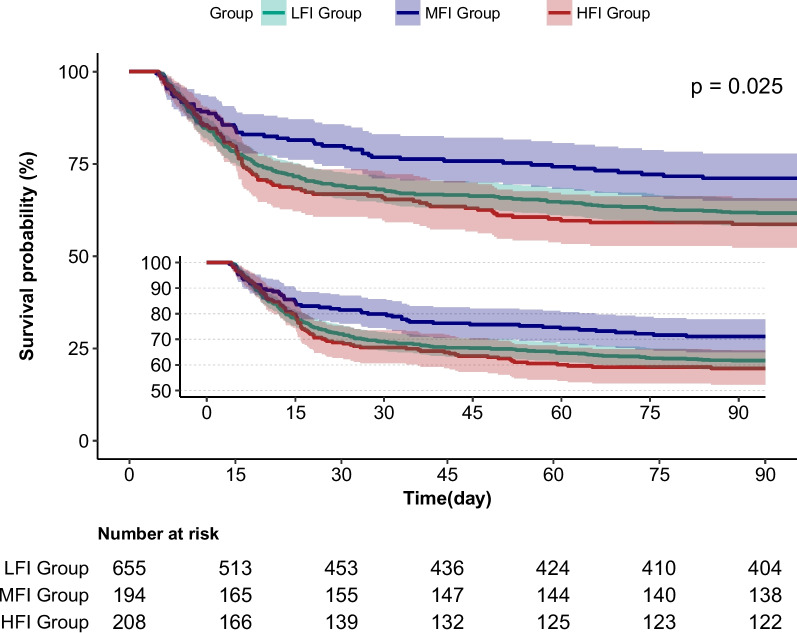
Kaplan–Meier survival curves for 90-day survival among groups

Log-rank test and Kaplan–Meier (KM) curves showed notable distinctions among three groups, with the MFI group demonstrating the highest survival rate compared to the other groups (*p* = 0.025) (see Fig. 3).

### Secondary outcomes and subgroup analysis

Compared to the LFI group, the MFI group exhibited the lowest hospital mortality following covariate adjustment, with an odds ratio (OR) of 0.62 (95% CI: 0.4 ~ 0.95, *p* = 0.028), while no statistically significant differences were observed in ICU mortality, length of hospital/ICU stay, duration of mechanical ventilation, incidences of ventilator-associated pneumonia, nosocomial infections, or Clostridium difficile infection, as presented in Table 4. Subgroup analysis, conducted without detecting any interactions between subgroups, consistently indicated the lowest 28-day mortality in the MFI group, as demonstrated in Supplementary sTable 2.

## Discussion

We analyzed 1057 septic patients receiving mechanical ventilation in the MICU or mixed ICU. We identified a J-shaped association between FI and 28-day mortality, with the lowest mortality observed within the 3 ~ 35 g/(%) FI range. The MFI group demonstrated significantly lower mortality rates at 28, 60, and 90 days, as well as in-hospital mortality when compared to the LFI group. Conversely, the HFI group showed no statistically significant differences in comparison to the LFI group. These trends persisted after adjusting for covariates. Nevertheless, no significant associations were found between FI and ICU mortality, length of hospital/ICU stay, or the risk of hospital-acquired infections, even after controlling for confounding factors.

In our study, it was found that 92.62% of mechanically ventilated septic patients received nutritional support via enteral nutrition, with the highest proportion observed in the MFI and HFI groups, significantly surpassing that of the LFI group. Additionally, the HFI group had the highest proportion of early (within 48 h) initiation of enteral nutrition at 80.288%, followed by the MFI group (52.577), while the LFI group had the lowest proportion (32.214%). This observation may be attributed to the more severe condition of the LFI group, as evidenced by higher SOFA scores and greater use of vasoactive agents (higher norepinephrine equivalents) within the first 72 h. Furthermore, we also observed that within the MFI group, the actual energy intake, protein intake, dietary fiber intake, dietary fiber index, and energy achievement percentage within the initial 72 h were the highest among the three groups.

Early enteral nutrition may potentially reduce the mortality rate in critically ill patients, and nutritional guidelines recommend early enteral nutrition for hemodynamically stable patients [8]. However, recent clinical research has yielded conflicting results regarding whether early enteral nutrition reduces mortality in critically ill patients, largely attributed to the risk of overfeeding when initiated prematurely [25–27]. In our study, the poorer prognosis in HFI group may be attributed to the reasons as follows: firstly, patients with a high fiber index were always associated with the adverse risks of overfeeding at the early stage. Secondly, high fiber itself may lead to feeding intolerance [28, 29] during the early stage.

In our study, we observed a positive correlation between dietary fiber intake and energy intake. Therefore, we calculated the dietary fiber index as a relative dose. Furthermore, in our multivariate analysis, we included actual energy intake as a confounding variable to adjust the model. Remarkably, our results remained consistent. This indicates that early dietary fiber intake (calculated using the dietary fiber index) independently influences the 28-day mortality rate in sepsis.

Dietary fiber (DF) comprises carbohydrates with degrees of polymerization (DP) ranging from 3 to 9, remaining undigested by the intestine while conferring physiological health benefits [30]. DF is commonly incorporated into enteral formulas and supplements to enhance intestinal motility and alleviate diarrhea in critically ill patients. Furthermore, research has indicated additional advantages [31] when DF is administered alone or as part of synbiotics to critically ill patients or animals, although limited investigations have focused on septic ICU patients. In septic animal experiments, high doses of DF administered before modeling not only mitigated the inflammatory response [32–35] but also decreased endotoxin-induced intestinal permeability [36, 37] and improved the survival of sepsis models [38]. Conversely, varying results have been reported in clinical studies [39, 40].

DF can improve mortality in critical patients depends on the dose [33] and timing [41] of DF administration. However, some studies have shown different outcomes. For example, a study involving mechanically ventilated septic patients receiving mixed DF for six days did not affect mortality or hospital stay [5]. In a randomized trial of 72 mechanically ventilated septic patients, one group received daily synbiotics (including galactooligosaccharides 10 g/day as prebiotics) starting from ICU admission, while the other received a placebo. Results showed significant differences in bacteremia incidence and 4-week mortality between the groups, with lower VAP incidence in the synbiotic group [14]. Knight et al. conducted a prospective, randomised, double blind, placebo controlled tria with 259 critically ill patients receiving mechanical ventilation for 48 h or longer, randomly assigning them to receive synbiotics (Betaglucan, Inulin, Pectin and Resistant starch (2.5 g of each) as prebiotics) or a placebo(cellulose). They found no differences in VAP incidence, VAP rate, or in-hospital mortality between the groups [42]. A smaller-scale study by Seifi et al. administered synbiotics (prebiotics: fructooligosaccharides) for 14 days to critically ill patients, resulting in reduced NLR and serum endotoxin levels but no differences in ICU outcomes [43]. Regarding VAP prevention, meta-analyses have shown mixed results. Some favor synbiotics over probiotics [12], while others suggest mixed probiotics are effective [13]. A meta-analysis by Liu et al. [33] found DF reduced C-reactive protein and hospital stay but had improved effects on ventilation duration and mortality only in the subgroup fed ≥ 20 g/d of DF. Although DF has been linked to reduced C. difficile infections in non-severe disease [44] and in animal study [45], however, our study did not find this association in critically ill septic patients. Our study found that early and medium dietary fiber intake can improve the mortality rate of septic patients. However, the specific types and dosages of dietary fiber require further confirmation through well-designed randomized controlled trials.

### Strengths and limitations

To the best of our knowledge, this is the first study to explore the relationship between DF and 28-day mortality, and identify the optimal amount of DF in mechanically ventilated patients with sepsis, especially in the early stages of sepsis. Current dietary fiber recommendations continue to rely on daily energy consumption [46]. Given the restricted energy intake during the early stages [47] of the highly catabolic phase of sepsis [48], coupled with variations in DF intake due to trophic and target feeding, we utilized the FI [17] as an indicator of early DF intake. This approach mitigates the impact of inconsistent energy intake, standardizes individual energy consumption, and enables somewhat meaningful comparisons. Additionally, we accounted for non-nutrient energy sources, such as dextrose and propofol. Furthermore, we adjusted more confounders such as age, BMI, SOFA, SAPSII, CCI, vasoactive agents, norepinephrine equivalents, feeding route, early enteral nutrition and actual energy intake. This study has limitations. We couldn’t access data on potential adverse effects of DF intake due to database constraints. Additionally, the effect of different kind of DF in the formula was not evaluated. Moreover, this is a retrospective study with numerous confounding factors, so more rigorously designed RCT studies are needed to confirm the impact of specific dietary fiber on clinical outcomes in early septic patients.

In conclusion, this retrospective cohort study found early (within 72 h of admission) optimal fiber intake (measured by FI, FI range from 3 to 35 g/(%)) can improve 28-day mortality in septic patients with invasive mechanical ventilation in the MICU or S/MICU.

### Supplementary Information

**Additional file 1: sTable 1.** Specifications and nutrients in MIMIC IV 2.1 Database.

**Additional file 2:** **sTable.** Subgroup analysis of 28-day mortality.

## Data Availability

The data analyzed was obtained from the Medical Information Mart for Intensive Care IV (MIMIC-IV) Clinical Database; the following licenses/restrictions apply: To access the data, you must be a credentialed user, complete the required training (CITI Data or Specimens Only Research) and sign the data use agreement for the project. Requests to access these datasets should be directed to PhysioNet, https://physionet.org/; https://physionet.org/content/mimiciv/2.1/.)

## Abbreviations

MICU: Intensive care unit
S/MICU: Surgical/Medical Intensive care unit
MIMIC-IV Database: Medical Information Mart for Intensive Care IV (MIMIC-IV) Clinical Database
DF: Dietary fiber
FI: Fiber index
SCFA: Short-chain fatty acid
EN: Enteral nutrition
PN: Parenteral nutrition
VAP: Ventilator-associated pneumonia
BMI: Body Mass Index
SOFA: Sequential Organ Failure Assessment
SASPII: Simplified Acute Physiology Score II
CCI: Charlson comorbidity index
PPI: Proton pump inhibitors
H_2_RA: H_2_ receptor antagonists
