# Supplementary material for: Association of early dietary fiber intake and mortality in septic patients with mechanical ventilation based on MIMIC IV 2.1 database: a cohort study
Source: Nutr J. 2024 Jan 3;23:1. doi: 10.1186/s12937-023-00894-1 (PMC10762999; doi:10.1186/s12937-023-00894-1)
Supplement: Supplementary file 2 — Additional file 2: sTable. Subgroup analysis of 28-day mortality. [file 12937_2023_894_MOESM2_ESM.docx]

| **sTable 2. Subgroup analysis of 28-day mortality** | | | | | | | | |
| --- | --- | --- | --- | --- | --- | --- | --- | --- |
| **Subgroup** | **N** | **Event (%)** | **Non-adjust Model** | | **Model 1** | | **P.for**  **Interaction 1** | **P.for**  **Interaction 2** |
|  |  |  | **HR (95%CI)** | **P value** | **HR (95%CI)** | **P value** |  |  |
| **Age（year）** | | |  |  |  |  | **0.806** | **0.930** |
| **＜65** |  |  |  |  |  |  |  |  |
| LFI Group n=655 | 337 | 76 (22.6) | 1(Ref) |  | 1(Ref) |  |  |  |
| MFI Group n=194 | 99 | 15 (15.2) | 0.66 (0.38~1.14) | 0.138 | 0.78 (0.43~1.39) | 0.394 |  |  |
| HFI Group n=208 | 99 | 19 (19.2) | 0.85 (0.52~1.41) | 0.536 | 1.07 (0.61~1.90) | 0.809 |  |  |
| **65~80** |  |  |  |  |  |  |  |  |
| LFI Group n=655 | 213 | 72 (33.8) | 1(Ref) |  | 1(Ref) |  |  |  |
| MFI Group n=194 | 68 | 16 (23.5) | 0.64 (0.37~1.11) | 0.11 | 0.61 (0.35~1.06) | 0.080 |  |  |
| HFI Group n=208 | 78 | 33 (42.3) | 1.27 (0.84~1.92) | 0.253 | 1.48 (0.92~2.38) | 0.105 |  |  |
| **≥80** |  |  |  |  |  |  |  |  |
| LFI Group n=655 | 105 | 51 (48.6) | 1(Ref) |  | 1(Ref) |  |  |  |
| MFI Group n=194 | 27 | 8 (29.6) | 0.56 (0.26~1.18) | 0.125 | 0.50 (0.23~1.10) | 0.086 |  |  |
| HFI Group n=208 | 31 | 17 (54.8) | 1.11 (0.64~1.92) | 0.716 | 0.97 (0.50~1.86) | 0.926 |  |  |
| **BMI (Kg/m^2^)** | | |  |  |  |  | 0.229 | 0.264 |
| **＜18.5** |  |  |  |  |  |  |  |  |
| LFI Group n=655 | 20 | 10 (50) | 1(Ref) |  | 1(Ref) |  |  |  |
| MFI Group n=194 | 7 | 2 (28.6) | 0.52 (0.11~2.36) | 0.395 | 0.19 (0.03~1.29) | 0.089 |  |  |
| HFI Group n=208 | 10 | 5 (50) | 0.88 (0.3~2.57) | 0.809 | 0.26 (0.05~1.29) | 0.099 |  |  |
| **18.5~25** |  |  |  |  |  |  |  |  |
| LFI Group n=655 | 181 | 62 (34.3) | 1(Ref) |  | 1(Ref) |  |  |  |
| MFI Group n=194 | 58 | 11 (19) | 0.52 (0.27~0.98) | 0.043 | 0.49 (0.26~0.95) | 0.035 |  |  |
| HFI Group n=208 | 59 | 20 (33.9) | 0.97 (0.59~1.6) | 0.902 | 1.05 (0.58~1.90) | 0.861 |  |  |
| **25~30** |  |  |  |  |  |  |  |  |
| LFI Group n=655 | 174 | 63 (36.2) | 1(Ref) |  | 1(Ref) |  |  |  |
| MFI Group n=194 | 53 | 11 (20.8) | 0.52 (0.27~0.98) | 0.044 | 0.44 (0.22~0.88) | 0.020 |  |  |
| HFI Group n=208 | 63 | 18 (28.6) | 0.73 (0.43~1.24) | 0.246 | 0.68 (0.37~1.25) | 0.215 |  |  |
| **≥30** |  |  |  |  |  |  |  |  |
| LFI Group n=655 | 280 | 64 (22.9) | 1(Ref) |  | 1(Ref) |  |  |  |
| MFI Group n=194 | 76 | 15 (19.7) | 0.86 (0.49~1.51) | 0.593 | 1.07 (0.60~1.90) | 0.857 |  |  |
| HFI Group n=208 | 76 | 26 (34.2) | 1.62 (1.03~2.55) | 0.038 | 2.04 (1.20~3.47) | 0.014 |  |  |
| **SOFA** | | |  |  |  |  | 0.904 | 0.711 |
| **＜6** |  |  |  |  |  |  |  |  |
| LFI Group n=655 | 70 | 17 (24.3) | 1(Ref) |  | 1(Ref) |  |  |  |
| MFI Group n=194 | 28 | 4 (14.3) | 0.57 (0.19~1.7) | 0.316 | 0.87 (0.26~2.88) | 0.818 |  |  |
| HFI Group n=208 | 28 | 5 (17.9) | 0.71 (0.26~1.94) | 0.507 | 1.58(0.45~ 5.50) | 0.475 |  |  |
| **≥6** |  |  |  |  |  |  |  |  |
| LFI Group n=655 | 585 | 182 (31.1) | 1(Ref) |  | 1(Ref) |  |  |  |
| MFI Group n=194 | 166 | 35 (21.1) | 0.64 (0.45~0.93) | 0.017 | 0.63 (0.43~0.91) | 0.014 |  |  |
| HFI Group n=208 | 180 | 64 (35.6) | 1.16 (0.87~1.54) | 0.304 | 1.19 (0.86~1.64) | 0.298 |  |  |
| **Feeding route** | | |  |  |  |  | 0.647 | 0.566 |
| **EN** |  |  |  |  |  |  |  |  |
| LFI Group n=655 | 593 | 184 (31) | 1(Ref) |  | 1(Ref) |  |  |  |
| MFI Group n=194 | 187 | 37 (19.8) | 0.6 (0.42~0.86) | 0.005 | 0.62 (0.44~0.89) | 0.009 |  |  |
| HFI Group n=208 | 199 | 66 (33.2) | 1.07 (0.8~1.41) | 0.654 | 1.15 (0.84~1.58) | 0.393 |  |  |
| **EN+PN** |  |  |  |  |  |  |  |  |
| LFI Group n=655 | 62 | 15 (24.2) | 1(Ref) |  | 1(Ref) |  |  |  |
| MFI Group n=194 | 7 | 2 (28.6) | 1.14 (0.26~4.99) | 0.862 | 2.75 (0.45~16.69) | 0.270 |  |  |
| HFI Group n=208 | 9 | 3 (33.3) | 1.47 (0.43~5.1) | 0.539 | 3.12 (0.62~15.78) | 0.169 |  |  |
| **Vasoactive agents used** | | |  |  |  |  | 0.648 | 0.917 |
| **No** |  |  |  |  |  |  |  |  |
| LFI Group n=655 | 182 | 42 (23.1) | 1(Ref) |  | 1(Ref) |  |  |  |
| MFI Group n=194 | 67 | 8 (11.9) | 0.5 (0.23~1.06) | 0.069 | 0.51 (0.23~1.13) | 0.097 |  |  |
| HFI Group n=208 | 80 | 19 (23.8) | 1.06 (0.61~1.82) | 0.844 | 1.31 (0.71~2.43) | 0.393 |  |  |
| **Yes** |  |  |  |  |  |  |  |  |
| LFI Group n=655 | 473 | 157 (33.2) | 1(Ref) |  | 1(Ref) |  |  |  |
| MFI Group n=194 | 127 | 31 (24.4) | 0.7 (0.48~1.03) | 0.072 | 0.68 (0.46~1.00) | 0.049 |  |  |
| HFI Group n=208 | 128 | 50 (39.1) | 1.19 (0.86~1.63) | 0.291 | 1.08 (0.76~1.55) | 0.873 |  |  |
| **Number of antibiotics** | | |  |  |  |  | 0.733 | 0.432 |
| **＜3** |  |  |  |  |  |  |  |  |
| LFI Group n=655 | 209 | 59 (28.2) | 1(Ref) |  | 1(Ref) |  |  |  |
| MFI Group n=194 | 58 | 12 (20.7) | 0.7 (0.38~1.31) | 0.266 | 0.71 (0.38~1.34) | 0.289 |  |  |
| HFI Group n=208 | 68 | 25 (36.8) | 1.33 (0.83~2.12) | 0.232 | 1.29 (0.74~2.24) | 0.362 |  |  |
| **≥3** |  |  |  |  |  |  |  |  |
| LFI Group n=655 | 446 | 140 (31.4) | 1(Ref) |  | 1(Ref) |  |  |  |
| MFI Group n=194 | 136 | 27 (19.9) | 0.6 (0.4~0.91) | 0.015 | 0.61 (0.40~0.93) | 0.022 |  |  |
| HFI Group n=208 | 140 | 44 (31.4) | 1 (0.71~1.4) | 0.981 | 1.13 (0.76~1.66) | 0.549 |  |  |
| Model 1: Adjust for age, BMI, SOFA, SAPSII, CCI, Vasoactive agents, Norepinephrine equivalents, feeding route, Early enteral nutrition and actual energy intake. Actual energy intake includes PN, EN and non-nutrient energy intake (dextrose and propofol).  Abbreviations: HR= hazard ratio, OR= odds ratio, CI= confidence interval, BMI= Body Mass Index, SOFA= Sequential Organ Failure Assessment. | | | | | | | | |
